# COVID-19 and Palliative Care Services: Comparative Patterns of Inpatient, Outpatient, and Consultation Services in a Tertiary Care Center in Riyadh

**DOI:** 10.7759/cureus.11996

**Published:** 2020-12-09

**Authors:** Maied Z AlShehery, Balaji Duraisamy, Abdul Rehman Z Zaidi, Nawal F AlShehry, Fatima Z Zaidi, Ahmad A Rababah, Abdulhakeem A Assiri, Mubarak S AlGhamdi, Abbas Al Mutair, Awad Al-Omari

**Affiliations:** 1 Department of Palliative Care, King Fahad Medical City, Riyadh, SAU; 2 Internal Medicine, Dr. Sulaiman Al-Habib Medical Group, Riyadh, SAU; 3 College of Medicine, Alfaisal University, Riyadh, SAU; 4 Department of Medicine, King Fahad Medical City, Riyadh, SAU; 5 Department of Adult Hematology, King Fahad Medical City, Riyadh, SAU; 6 Medical Education, University College of Medicine and Dentistry, University of Lahore, Lahore, PAK; 7 Physiology, University of Health Sciences, Lahore, PAK; 8 Hematology and Oncology Center, Dr. Sulaiman Al-Habib Medical Group, Riyadh, SAU; 9 Nursing, Wollongong University, New South Wales, AUS; 10 Research Center, Almoosa Specialist Hospital, Al-Ahsa, SAU; 11 Research Center, Dr. Sulaiman Al-Habib Medical Group, Riyadh, SAU

**Keywords:** coronavirus disease 2019 (covid-19), sars-cov-2 (severe acute respiratory syndrome coronavirus -2), covid-19, palliative care, palliative and supportive care, impact on service, practice patterns

## Abstract

Introduction

The coronavirus disease 2019 (COVID-19) outbreak caused by the severe acute respiratory syndrome coronavirus 2 (SARS-CoV-2) marked the third introduction of a highly pathogenic and large-scale epidemic coronavirus into the human population in the 21st century. The World Health Organization declared the COVID-19 outbreak as a pandemic on March 11, 2020. Lockdowns were imposed in multiple countries affecting patient flow in hospitals.

Methods

This is a retrospective study conducted at King Fahad Medical City (KFMC), a tertiary care hospital in Riyadh, Saudi Arabia, which examined the differences in palliative care services during the initial four months of the COVID-19 pandemic compared to the respective four months in 2019 (March, April, May, June).

Results

A total of 319 patients were seen at the palliative care department from March to June 2020 during the COVID-19 pandemic (119 inpatient, 200 outpatient), compared to 346 patients seen during the corresponding months in 2019 (97 inpatient, 249 outpatient). Our main findings included more patients being discharged home, lesser transfers, shorter hospital length of stay, lesser imminent death protocols, and a higher palliative performance score (PPS) during the COVID-19 pandemic. Although there were more cancelations by the hospital for the outpatient department, a virtual clinic was started, and 84 patients were effectively seen. Around 87% of patients were fully satisfied (5/5) with the services provided by the virtual clinic. There were no positive COVID-19 cases in our healthcare workers in the palliative care department due to the high standard precautions applied at KFMC. Family meetings as well as administrative and academic meetings have been efficiently held virtually and may possibly become the standard of practice.

Conclusion

Palliative care services were successfully maintained during the COVID-19 pandemic at KFMC.

## Introduction

Palliative care is an emerging subspecialty in medicine that focuses mainly on patients affected by chronic life-threatening illnesses that will eventually interfere with their life expectancy and well-being [1]. Palliative care aims to aid patients and their families during the progression of the disease and ease the patient’s suffering caused by that chronic disease. The World Health Organization (WHO) has recognized palliative care as a basic human right. Moreover, approximately 40,000,000 people require palliative care services, including ~5,500,000 patients with terminal cancer, various of who suffer from moderate to severe pain, without having access to appropriate opioid pain medications. Approximately 14% of the public who require palliative care services receive it [2].

A comprehensive multidisciplinary approach is utilized where all possible impacts of a chronic, incurable disease are taken into account and appropriate symptom relief is provided. It encompasses the psychological, spiritual, and social dimensions in addition to physical symptoms that complicate a chronic, life-shortening disease [3]. When cancer progresses to a stage that it is no longer responsive to treatment, the chances of improving survival is limited. However, we can still improve the quality of life of patients and their families by ameliorating their symptoms using palliative care [3]. The cancer prevalence rate is universally increasing in the West and Saudi Arabia, which may be imputed to the lifestyle and demographics of the public. Although the WHO has accepted palliative care as a basic human right, in Saudi Arabia, only secondary and tertiary care hospitals can provide it. Although the cancer rate in Saudi Arabia has been projected to go higher in the next few years, the expansion of palliative care services in Saudi Arabia has not been as rapid.

The coronavirus disease 2019 (COVID-19) pandemic carries on to surge around the world and has caused consequential disruption of multiple healthcare services. There have been several measures taken worldwide to control COVID-19 and secure continuity of urgent healthcare services, cancer care being one of them. In Saudi Arabia, the first case of COVID-19 was found entering its border on March 2, 2020. As of September 10, 2020, there have been over 322,000 confirmed cases with approximately 4,100 deaths in the Kingdom [4]. In March, the government-imposed lockdowns and curfews to limit the spread of COVID-19, and offenders were fined heftily to ensure strict compliance. Along with several aspects of life, the lockdown also had an impact on hospital services. The traffic through the hospital was generally reduced for most departments, possibly due to patients’ apprehension of contracting COVID-19. This study was conducted to examine the differences in palliative care services during the initial four months of the COVID-19 pandemic lockdown compared to the respective four months of 2019 (March, April, May, June).

## Materials and methods

Study design, participants, and study setting

This is a retrospective descriptive study conducted from July 2020 to August 2020. Medical records of patients under the care of the palliative care department (inpatient and outpatient) from March, April, May, and June 2019 (regular pre-COVID-19 era) and March, April, May, and June 2020 (during the COVID-19 pandemic lockdown) were reviewed for frequencies of no shows in the Outpatient Department (OPD), number of admissions, the length of stay of patients, number of patients referred to the palliative care department, number of patients transferred to palliative care, and the differences of diagnoses.

We divided the data into two groups for comparison, group 1: March, April, May, June 2019, and group 2: March, April, May, June 2020. Corresponding months of both years were selected to strengthen the study and decrease the disparity in data as there are seasonal changes in patterns of admissions, outpatient visits, and hospital logistics. This will give us a more accurate representation of the effect of COVID-19 on the palliative care department.

Ethical statement

This study was approved by the King Fahad Medical City Institutional Review Board, IRB Log No. 20-486 on July 23 2020. As this was a retrospective study, no informed consent was necessary, and data were de-identified for the use of this publication. This study adheres to the ethical guidelines of the Declaration of Helsinki and good clinical practice.

Statistical analysis

The statistical analysis was performed using Statistical Package for Social Sciences (SPSS) version 24 (IBM Corp., Armonk, NY, USA). Descriptive statistics were computed for each variable. We presented continuous variables as mean (SD) and categorical variables as number (%). We compared proportions for categorical variables between groups using the Chi-square test. We compared means of continuous variables between groups using independent group t-tests when values were normally distributed; for non-normal data, we used the Mann-Whitney U test.

## Results

A total of 319 patients were seen at the palliative care department from March to June 2020 during the COVID-19 pandemic, compared to 346 patients seen during the corresponding months in 2019. One hundred nineteen patients were inpatient and 200 were outpatient in 2020, in contrast to 97 inpatient patients and 249 outpatient patients seen during the equivalent months in 2019.

The mean age of patients seen in the inpatient services in 2019 was 64 ± 16.44 and 59.28 ± 17.66 years in 2020. More females and Saudis were generally seen during both years. There were more palliative care unit (PCU) admissions and relatively more carried over patients during 2020. The top diagnosis/reason for admission of patients seen in 2020 was End of Life, 10% more than in 2019. 

During COVID-19, there were more old cases than new cases (p = 0.03) in the ward. The length of stay of patients at the hospital was also reduced by an average of two days (p = 0.3). More patients were carried over or discharged home, and lesser patients were transferred (p = 0.054) due to the high risks involved in transferring patients during the pandemic. The number of imminent death protocols (IDPs) was also lesser this year than the past year (p=0.003). Statistics of inpatient services in the pre-COVID-19 and the COVID-19 era are detailed in Table 1 and Table 2.

**Table 1:** Statistics of Inpatient Services in the pre-COVID-19 and COVID-19 era *many centers do not have this protocol adapted by King Fahad Medical City PPS = palliative performance score, PCU = palliative care unit, IQR = interquartile range, DNR = do not resuscitate, DAMA = discharge against medical advice

Parameter	2019 (Mar-Jun) N= 97	2020 (Mar-Jun) N= 119	P-value
Age			0.044
Mean ± SD	64 ± 16.438	59.28 ± 17.664	
Min-Max	16-96	21-100	
Gender			0.672
Male	38 (39.2%)	50 (42%)	
Female	59 (60.8%)	69 (58%)	
Nationality			0.419
Saudi	96 (99%)	116 (97.5%)	
Non-Saudi	1 (1%)	3 (2.5%)	
Admission Type			0.443
PCU Admission	25 (25.8%)	39 (32.8%)	
Transferred to PCU	62 (63.9%)	66 (55.5%)	
Carried over	10 (10.3%)	14 (11.8%)	
Admitted From			0.142
ER	23 (23.7%)	38 (31.9%)	
GI	1 (1%)	0 (0%)	
Hematology	1 (1%)	0 (0%)	
Maternity	3 (3.1)	2 (1.7%)	
Medical	2 (2.1%)	4 (3.4%)	
Medical Oncology	54 (55.7%)	60 (50.4%)	
Neuroscience	6 (6.2%)	4 (3.4%)	
Radiation	2 (2.1%)	3 (2.5%)	
Surgical	5 (5.2%)	8 (6.7%)	
Code Status			
DNR	97 (100%)	119 (100%)	
Specialty			0.648
GI	2 (2.1%)	2 (1.7%)	
Hematology	1 (1%)	6 (5%)	
Maternity	2 (2.1%)	1 (0.8%)	
Medical	1 (1%)	3 (2.5%)	
Neuroscience	6 (6.2%)	4 (3.4%)	
Oncology Palliative	48 (49.5%) 26 (26.8%)	57 (47.9%) 36 (30.3%)	
Radiation	6 (6.2%)	5 (4.2%)	
Surgical	5 (5.2%)	5 (4.2%)	
Top Diagnosis			0.590 (LR)
Cancer Pain	26 (26.8%)	24 (20.2%)	
Delirium	3 (3.1%)	3 (2.5%)	
Dyspnea	7 (7.2%)	5 (4.2%)	
End of Life	57 (58.8%)	82 (68.9%)	
GI Symptoms	4 (4.1%)	5 (4.2%)	
New Case	70 (72.2%)	69 (58.0%)	0.030
Old Case	27 (27.8%)	50 (42.0%)	
PPS			
Median (IQR)	30 (20-40)	40 (30-40)	0.001
Length of Stay			0.311
Median (IQR)	10 (4-19)	8 (4-19)	
Discharge Type			0.054
Carried Over	19 (19.6%)	28 (23.5%)	
Discharged Home	24 (24.7%)	43 (36.1%)	
Passed Away	42 (43.3%)	44 (37.0%)	
Transferred	11 (11.3%)	4 (3.4%)	
DAMA	1 (1.0%)	0 (0.0%)	
Opioids			
Yes	97 (100%)	119 (100%)	
No	0 (0%)	0 (0%)	
Laxatives			
Yes	97 (100%)	119 (100%)	
No	0 (0%)	0 (0%)	
Imminent Death Protocol *			0.003
Yes	17 (17.5%)	6 (5.0%)	
No	80 (82.5%)	113 (95.0%)	

**Table 2:** Inpatient Diagnoses in 2019 and 2020 NSCLC = non-small cell lung cancer, SCC = squamous cell carcinoma, ALL = acute lymphocytic leukemia, AML = acute myeloid leukemia, GBM = glioblastoma

Diagnosis	2019 (Mar-Jun) N= 97	2020 (Mar-Jun) N= 119
Acute Leukemia AML/ALL	2 (2.1%)	1 (0.8%)
Appendicular Cancer	1 (1.0%)	1 (0.8%)
Astrocytoma	0 (0.0%)	5 (4.2%)
B Cell & Burkitt Lymphoma	0 (0.0%)	2 (1.7%)
Bladder Cancer	8 (8.2%)	0 (0.0%)
Breast Cancer	14 (14.4%)	13 (10.9%)
Breast Cancer and Endometrial Cancer	1 (1.0%)	0 (0.0%)
Cervical Cancer	0 (0.0%)	2 (1.7%)
Cholangiocarcinoma	1 (1.0%)	4 (3.4%)
Colorectal Cancer	10 (10.3%)	11 (9.2%)
Endometrial Cancer	4 (4.1%)	2 (1.7%)
Esophageal Cancer	2 (2.1%)	7 (5.9%)
Ewing sarcoma	1 (1.0%)	0 (0.0%)
Fibrosarcoma	0 (0.0%)	1 (0.8%)
Gallbladder Cancer	4 (4.1%)	2 (1.7%)
Gastric Cancer	2 (2.1%)	3 (2.5%)
GBM	10 (10.3%)	3 (2.5%)
Glioma	0 (0.0%)	1 (0.8%)
Hepatocellular Cancer	7 (7.2%)	7 (5.9%)
Laryngeal Cancer	1 (1.0%)	0 (0.0%)
Epithelioid	0 (0.0%)	1 (0.8%)
Lung Cancer Malignant Melanoma	4 (4.1%) 1 (1.0%)	3 (2.5%) 0 (0.0%)
Medulloblastoma	2 (2.1%)	4 (3.4%)
Meningioma	0 (0.0%)	2 (1.7%)
NSCLC Mets to brain	0 (0.0%)	1 (0.8%)
Multiple Myeloma	0 (0.0%)	6 (5.0%)
Myelofibrosis	0 (0.0%)	2 (1.7%)
Nasopharyngeal Cancer	1 (1.0%)	0 (0.0%)
Neuroendocrine Tumor	1 (1.0%)	0 (0.0%)
Neurofibromatosis	0 (0.0%)	1 (0.8%)
Oligodendroglioma	0 (0.0%)	1 (0.8%)
Osteosarcoma	2 (2.1%)	0 (0.0%)
Ovarian Cancer	2 (2.1%)	2 (1.7%)
Pancreatic Cancer	6 (6.2%)	5 (4.2%)
Parotid Cancer	1 (1.0%)	0 (0.0%)
Prostate Cancer	0 (0.0%)	3 (2.5%)
Pseudomyxoma Peritoni	1 (1.0%)	1 (0.8%)
Rectal Periampillary Cancer	1 (1.0%)	1 (0.8%)
Rectosigmoid Cancer	1 (1.0%)	2 (1.7%)
Rectum Cancer	3 (3.1%)	2 (1.7%)
Renal Cell Cancer	0 (0.0%)	8 (6.7%)
Sarcoma	0 (0.0%)	1 (0.8%)
SCC in the mouth	0 (0.0%)	2 (1.7%)
Tongue Cancer	1 (1.0%)	1 (0.8%)
Thyroid Cancer	1 (1.0%)	1 (0.8%)
Urinary Bladder Cancer	0 (0.0%)	1 (0.8%)
Uterine Cancer	1 (1.0%)	2 (1.7%)
Uterine Cancer/Cervical Cancer	0 (0.0%)	1 (0.8%)
P value = 0.027		

During the COVID-19 months of March, April, May, and June, a total of 200 patients were seen, unlike 2019 where 249 outpatient patients were taken care of. Out of the 200 patients, 84 were seen in the newly established virtual clinics. Out of the 84 patients seen virtually, 79 were follow-up patients and five were seen for the first time. The virtual clinics provided high satisfaction among patients as evaluated by a mini feedback survey. This survey asked the patients 1) if the virtual clinic covers all their needs and 2) how they would rate the overall palliative care clinic service. 51/84 completed the survey. 45/51 (88.23% people) answered 5/5 to whether the virtual clinic covered all their needs, while the remaining 6/51 (11.77% people) answered 4/5. For how they would rate the overall palliative care clinic service, 44/51 (86.27%) answered 5/5 and the remaining 7/51 (13.73%) answered 4/5.

The mean ± SD age for outpatient patients was 57.96 ± 17.25 and the median (IQR) PPS was 60 (50-70). There were 18 appointments in the OPD canceled by the hospital in 2020. Palliative care outpatient statistics are described in Table 3 and Table 4.

**Table 3:** Palliative Care Outpatient Statistics PPS = palliative performance score, DNR = do not resuscitate

		2019 (March-June) N=249	2020 (March-June) N=200	P-value
Age		57.92 ± 17.59	58.03 ± 16.83	0.949
Gender	Male	90 (36.1%)	72 (36%)	0.975
	Female	159 (63.9%)	128 (64%)	
Attendance	Show	150 (60.2%)	127 (63.5%)	0.0001
	No-Show	99 (39.8%)	55 (27.5%)	
	Cancelled by hospital	0 (0%)	18 (9%)	
Code Status	DNR	78 (31.3%)	73 (36.5%)	0.116
	Full Code	167 (67.1%)	127 (63.5%)	
	N/A	4 (1.6 %)	0 (0%)	
PPS		59.61 ± 13.65	60.46 ±13.39	0.681

**Table 4:** Outpatient Diagnoses in 2019 and 2020 AML = acute myeloid leukemia, DLBCL = diffuse large B-cell lymphoma, SCC = squamous cell carcinoma

Diagnosis	2019 (March-June) N=249	2020 (March-June) N=200
AML	1 (0.4%)	1 (0.5%)
Bladder Cancer	7 (2.8%)	2 (1%)
Breast Cancer	49 (19.7%)	35 (17.5%)
Colon Cancer	15 (6%)	14 (7%)
Cervical Cancer	2 (0.8%)	5 (2.5%)
Cholangiocarcinoma	0 (0%)	2 (1%)
Gall Bladder Cancer	3 (1.2%)	4 (2%)
Endometrial Cancer	7 (2.8%)	1 (0.5%)
Esophageal Cancer	1 (0.4%)	0 (0%)
Pancreatic Cancer	8 (3.2%)	6 (3.0%)
Ependymoma spine	3 (1.2%)	2 (1%)
Gastric Cancer	5 (2%)	5 (2.5%)
Glioblastoma	11 (4.4%)	0 (0%)
Hard palate adeno Cancer	3 (1.2%)	1 (0.5%)
Ovarian Cancer	2 (0.8%)	3 (1.5%)
Lung Cancer	13 (5.2%)	13 (6.5%)
Hepatocellular Cancer	8 (3.2%)	9 (4.5%)
Laryngeal Cancer	1 (0.4%)	0 (0%)
Meningioma	8 (3.2%)	4 (2%)
Hodgkins Lymphoma	1 (0.4%)	1 (0.5%)
Uterine Cancer	2 (0.8%)	5 (2.5%)
Thyroid Cancer	13 (5.2%)	7 (3.5%)
Medulloblastoma	4 (1.6%)	0 (0%)
Malignant Melanoma	1 (0.4%)	2 (1%)
Multiple Myeloma	6 (2.4%)	4 (2%)
Astrocytoma	1 (0.4%)	2 (1.0%)
DLBCL (Lymphoma)	6 (2.4%)	3 (1.5%)
Meningioma	1 (0.4%)	1 (0.5%)
Mesothelioma	2 (0.8%)	0 (0%)
Glioma	7 (2.8%)	6 (3.0%)
Rectosigmoid Cancer	4 (1.6%)	16 (8.0%)
Rectum Cancer	20 (8.0%)	4 (2.0%)
Nasopharyngeal Cancer	1 (0.4%)	6 (3%)
Prostate Cancer	4 (1.6%)	5 (2.5%)
Tonsillar Cancer	0 (0%)	1 (0.5%)
Sarcoma	8 (3.2%)	4 (2.0%)
SCC in the mouth	2 (0.8%)	3 (1.5%)
Schwanoma	2 (0.8%)	1 (0.5%)
Renal Cell Cancer	0 (0%)	3 (1.5%)
Tongue Cancer	1 (0.4%)	1 (0.5%)
Pseudomyxoma Peritoni	1 (0.4%)	2 (1.0%)
Neuralgia/ Scrambler therapy	1 (0.4%)	3 (1.5%)
Sickle Cell Disease	0 (0%)	1 (0.5%)
Myelofibrosis	1 (0.4%)	2 (1%)
Lumbar facet disease	0 (0%)	1 (0.5%)
Maxillary Cancer	2 (0.8%)	0 (0%)
Buccal Cancer	0 (0%)	1 (0.5%)
Lip Cancer	1 (0.4%)	0 (0%)
Non Polyposis Coli	0 (0%)	4 (2%)
Adenocarcinoma of unknown primary	4 (1.6%)	2 (1%)
Giant Cell Tumor	0 (0%)	1 (0.5%)
Germ Cell Tumor	2 (0.8%)	0 (0%)
Juvenile Xanthogranuloma	0 (0%)	1 (0.5%)
Trophoblastic Disease	1 (0.4%)	0 (0%)
Wrong Consultation	3 (1.2%)	0 (0%)
P-value = 0.0001		

## Discussion

Palliative care intends to subdue pain and other symptoms to ensure patients are as comfortable as possible while preserving emotional, spiritual, and social support for them and their families. Although the majority comprises cancer patients, plenty of chronic diseases patients have equivalent rights to receive such holistic end of life care. Cancer continues to grow and utilize healthcare resources across the Kingdom, with women having a higher frequency of cancer. The total annual cancer prevalence in Saudi Arabia was ~17,000 in 2016, with the age-standardized incidence rate of 91.3 per 100,000 in women versus 74.7 in men [5].

During the early days of lockdown, the admission rate decreased, mainly due to the depletion of hospital inflow. The overall reduction in bed occupancy of the hospital also resulted in lesser consultation requests for palliative care. However, the total admissions to the palliative care unit did not decrease over these four months. This may be due to the fact that primary care at our institution was closed during COVID-19 (to increase COVID-19 screening) which was counterproductive and this could be one reason why the number of admissions increased.

To ensure efficient infection control, multiple measures were taken to secure the spread of COVID-19 in the palliative department. Healthcare workers and patients were screened and tested for COVID-19. They were also educated about hand hygiene, mask etiquette, physical distancing, cough etiquette, and correct use of personal protective equipment along with other methods on how to prevent the spread of the virus. Our administration also quickly ensured that quick decisions and actions were taken to contain COVID-19 and prevent cross-infection.

Alsirafy et al. in 2009 reported 53.6% female versus 46.4% males in palliative care patients admitted over four years [6]. Accordingly in our study, 58-61% admitted patients were females as compared to 39-42% males, and a similar pattern of 64% females vs 36% males was witnessed in the patients presenting in the outpatient clinics. On the contrary, Robinson et al. reported a higher percentage of male patients (50.9%) as compared to female patients (49.1) admitted to the hospital for palliative care [7]. The gender distribution in our patients also differed from Verhoef et al. who reported 54.5% males and 45.5% females. The mean age of patients seen in our inpatient services in 2019 was 64 ± 16.44, which is similar to the median age (63 years) of patients needing palliative care observed by Verhoef et al. [8]. The mean age of inpatients in our study significantly differed between the years, being 59.28 ± 17.66 years in 2020. Robinson et al. reported 54.3% of palliative care admissions to fall within the age range of 60 to 79 years, followed by 26.7% of patients aged between 18 to 59 years and 19% aged >80 years [7]. The median age reported by Alsirafy et al. was 52 [6].

The majority of palliative care requiring admissions are cancer patients as reported to be 81.9% by Robinson et al. vs 18.1% non-cancer patients [7]. As our palliative care center is a part of the comprehensive cancer center, our patients were mostly cancer patients with breast cancer, endometrial cancer, Ewing sarcoma, laryngeal cancer, parotid cancer, glioma, renal cell carcinoma, sarcoma, and myelofibrosis as the top diagnoses. Our findings differed from those of Robinson et al. who had reported the highest percentage of lung cancer patients followed by breast cancer and gastrointestinal cancers [7]. Our findings also differed from those of the Australian Institute of Health and Welfare who reported 29.6% admissions due to pancreatic cancer, followed by lung cancer (28.1%) and liver cancer (21.6%) [9]. This shows that even though cancer predominates as the primary diagnosis of the admissions requiring palliative care, but as the geographic distribution of the population differs, the types of cancers vary.

Half or more of the patients admitted in the PCU are transferred from medical oncology, followed by a larger number of patients presenting to the emergency department for various symptoms. Transfers from other specialties were not significant. In our study, the top reasons for admission were similar in both groups/years with more than half of patients admitted for end-of-life care, followed by cancer pain. Verhoef et al. also reported pain as the second most frequent symptom in cancer patients admitted for palliation [8]. All our patients received opioids and laxatives.

In Australia, the average length of stay (ALOS) for palliative care patients is reported to be 10.6 days, and this number is considered to be about four times longer than the average LOS of 2.8 days for all hospitalizations [9]. In our population, the median LOS of palliative care patients in 2019 (before COVID-19) was similar to the LOS reported by them. However, the median hospital stay was reduced from 10 to eight days in 2020. Although not statistically significant, the decrease can be attributed to the apprehension caused by COVID-19. The decreased ALOS could be due to patients getting admitted with better performance status and so, were more likely to be discharged.

Palliative performance score (PPS) is an indicator used to document functional status and for tracking change over time. In our study, the median PPS at the time of admission statistically increased from 30 in the year 2019 to 40 in the year 2020. This indicates that patients under palliative care in 2020 were less likely to die than in 2019. Prognostication and diagnosis of imminent death is an important aspect of palliative care. During the pandemic, the number of patients with imminent death diagnosis is significantly less. This could be due to a high degree of suspicion of acute presentations of patients as COVID as opposed to disease progression. Imminent death diagnosis is ideally made by meticulous assessment/reassessment by a multidisciplinary team. The imminent death protocol was developed for very sick patients who were expected to survive for less than two weeks. Strict protocols of isolation and restricted access to a multidisciplinary team for assessment could also be factors leading to lower imminent death diagnosis during the pandemic [10].

All our inpatients had a do not resuscitate (DNR) code status; the majority of them passed away in 2019. However, this year a similar number of patients were discharged home. This may be attributed to a lesser number of imminent death protocols (IDPs) this year than the past year (p=0.003), which itself may be due to the non-availability of the multidisciplinary team in initiating the IDPs.

The number of outpatients dropped by a huge margin during the lockdown mainly due to the interstate and intrastate travel restrictions and governmental advisory of avoiding visits to the hospitals for non-urgent reasons. During the COVID-19 lockdown months of March, April, May, and June, a total of 200 patients were seen in the outpatient clinics, unlike in 2019 where 249 outpatient patients were taken care of. Out of the 200 patients, 84 were seen in the newly established virtual clinics, which means only 116 patients were seen in the hospital clinic. Compared to 249 in 2019, that is a drop by a huge margin. Another reason could be the fear of contracting COVID-19 [11]. Out of the 84 patients seen virtually, 79 were follow-up patients and five were seen for the first time. There were similar percentages of DNR and full code patients in 2019 and 2020.

To reduce the hospital visits of the patients and reduce the crowd in the hospital to avoid unnecessary exposure, we changed the departmental protocol and practices. The teleconsultations increased. The medication was issued for longer durations and medication via courier services was offered. In the absence of fresh complaints, patients were discouraged to visit the hospital during lockdown just for the sake of getting medicine refills. Other than virtual clinics and family/caregiver meetings over the phone, our general departmental practices also changed. While a few activities were canceled, administrative meetings were held online over platforms like Zoom. We also held academic/training activities virtually, where the residents and fellows outside the hospital premises could participate easily.

It seems Saudi Arabia was better prepared for this pandemic due to experience with the Middle East respiratory syndrome coronavirus (MERS-CoV), which was first identified in 2012 and caused various human-to-human outbreaks between 2014 and 2015. The MERS-CoV breakout resulted in the formation of several public health agencies including the Saudi Center for Disease Prevention and Control, the Command and Control Centre, and the National Health Laboratory. Moreover, the MERS-CoV experience contributed to the implementation of superior infection control procedures across hospitals in the Kingdom and finer coordination of care for impuissant cancer patients.

Continuity of care was a high priority in the Kingdom; the Saudi Arabia National Cancer Institute issued guidelines for hospitals and healthcare workers to help curtail the repercussions of COVID-19 on cancer patients. The guidelines included COVID-19 symptoms, guidance to guarantee prophylactic infection control settings, advice for establishing virtual clinics, and equipped all healthcare workers and cancer patients with permits to travel to the hospital during curfew/lockdown. Healthcare workers were directed to have virtual team meetings, to prioritize only urgent surgeries, and see patients virtually as much as possible. Cancer patients were provided with supportive information on an online portal and their medications were dispensed via mail or a drive-through pharmacy. The Middle East has shown effective telehealth use during the COVID-19 pandemic. Due to curfew and fear of contracting COVID-19, patients were reluctant to visit the hospital [11].

According to the literature, the key factors for patient satisfaction are the time spent by a patient waiting in the outpatient, how long and thoroughly he/she was seen by the physician, the clarity and quality of patient education, and perceived understanding [12]. Our survey results showed high percentages of satisfaction by the patients who visited the virtual clinics, indicating that these factors were not compromised. Patients were able to receive their desired levels of physician’s attention, time, adequate information, and medication.

This study had various strengths but also had several limitations. Firstly, our study was a cross-sectional study and because the pandemic of COVID-19 is not fully over yet, we could not assess the full-time period of the pandemic and match it to before the pandemic. However, this does provide a good insight into how we can successfully tackle and adjust our services during the acute phases of a future pandemic. Secondly, we could not analyze validated scales like the Edmonton Symptom Assessment System (ESAS) score, for symptom presence and intensity for the purpose of this study. Nevertheless, there are many lessons yet to be learned from the COVID-19 pandemic, and additional studies will benefit healthcare administrators and practitioners in planning ahead on how to preserve ongoing services in palliative care throughout the world.

## Conclusions

In conclusion, palliative care services were successfully maintained during the COVID-19 pandemic at our institution utilizing virtual clinics and implementing multiple infection control procedures in the hospital. Further collaborative studies need to be done, especially in our region, to be well prepared for future pandemics.
